# County-level differences in *Salmonella* incidence across urban and rural United States, 2011–2023

**DOI:** 10.1017/S0950268826102192

**Published:** 2026-10-14

**Authors:** Lavin A. Joseph, Lyndsay Bottichio, Ethel V. Taylor, Grace Bagwell Adams, Suhang Song

**Affiliations:** 1 Department of Health Policy and Management, University of Georgia, USA; 2 Centers for Disease Control and Prevention, USA

**Keywords:** NNDSS, Salmonella, Rural, Urban, Decomposition

## Abstract

*Salmonella* is a leading cause of foodborne illness in the United States, yet rural–urban differences in incidence and their underlying drivers remain poorly characterized. We conducted an ecological analysis of deidentified probable and confirmed *Salmonella* cases from the National Notifiable Diseases Surveillance System (NNDSS), 2011–2023, to evaluate temporal, regional, and population-level determinants of *Salmonella* incidence. Multivariable negative binomial models assessed rural–urban differences nationally and by U.S. Census region, and Blinder–Oaxaca decomposition quantified contributions of demographic, socioeconomic, healthcare access, and health-related factors. Age-adjusted incidence was consistently higher in rural than in urban counties. After adjustment, rural counties had higher incidence nationally and separately in all four regions (Northeast, Midwest, West, and South). County-level factors explained 66% of the national rural–urban gap, driven primarily by age structure, income, education, and food environment. Adults aged ≥65 years accounted for approximately 60% of the explained difference and were the dominant contributor in some regions. Regional variation was evident, with age predominating in the Midwest and West and education contributing more in the Northeast and South. A substantial unexplained component remained. These findings highlight persistent, regionally heterogeneous disparities and underscore the need for targeted, region-specific surveillance and prevention strategies.

## Introduction

Non-typhoidal *Salmonella* is a leading cause of foodborne illness in the United States, resulting in approximately 1.28 million illnesses, 12 500 hospitalizations, and 238 deaths annually [1, 2]. Most of these infections are self-limiting; however, life-threatening illnesses are most common among young children, older adults, and immunocompromised persons [3, 4]. *Salmonella* incidence is affected by individual and community-level determinants, including demographic composition, socioeconomic factors, and underlying health status [5–9].

The relationship between rural–urban differences in *Salmonella* incidence is heterogeneous across studies and geographic contexts. Some studies have indicated that rural areas have higher incidence of *Salmonella* Javiana, Newport, and Typhimurium serotypes, while environmental factors have been shown to influence salmonellosis risk and may confound observed rural–urban patterns [10]. Earlier national surveillance analyses suggested lower *Salmonella* incidence in more urban counties after adjustment for population characteristics [5]. Subsequent laboratory-based analyses indicated a higher probability of case detection in urban counties, suggesting that rural–urban differences may partly reflect variation in case detection rather than true differences in per-capita incidence [8]. State-level studies have yielded conflicting results, with no differences between rural and urban areas identified in the midwestern state of Michigan [11–13]. However, analysis using data from the southern state of Florida has highlighted associations between *Salmonella* incidence and population density–related characteristics, reflecting contextual factors associated with rural–urban structure [14].

Understanding rural–urban differences in *Salmonella* incidence aligns with Healthy People 2030 priorities to reduce disparities and improve population health through targeted interventions addressing healthcare access, social determinants, and environmental conditions [15]. Examining how *Salmonella* incidence differs between rural and urban areas is, therefore, important not only for informing targeted prevention strategies but also for identifying inequities in risk and surveillance that may hinder progress toward these national goals. These rural-urban differences could develop from variations in population attributes, income and education, healthcare access, and overall health, though the extent to which each factor contributes is unclear. In addition, few studies have determined whether rural–urban differences in *Salmonella* incidence persist after adjustment for these county-level characteristics, particularly across U.S. Census regions using models appropriate for overdispersed count data. Measuring the influence of these factors will help to identify which population attributes are responsible for the most significant disparities. Even though *Salmonella* incidence is monitored through national surveillance systems, recent national assessments of urban-rural disparities are limited. To address these gaps, we used the National Notifiable Diseases Surveillance System (NNDSS) data from 2011 to 2023 to (1) characterize temporal and regional patterns in county-level, age-adjusted *Salmonella* incidence across urban and rural areas, (2) estimate adjusted rural–urban differences overall and by Census region, and (3) quantify the relative contributions of population characteristics, including demographic, socioeconomic, healthcare access, and health-related factors to observed disparities overall and by Census region.

## Methods

### Study design and data sources

This ecological study examined differences in county-level *Salmonella* incidence between rural and urban counties in the United States using de-identified probable and confirmed case data from NNDSS from January 1, 2011, through December 31, 2023. Data were accessed through the CDC’s System for Enteric Disease Response, Investigation, and Coordination (SEDRIC) [16]. NNDSS data are publicly available from the CDC at https://www.cdc.gov/nndss/infectious-disease [17]. Case status (confirmed or probable) was defined according to the Council of State and Territorial Epidemiologists (CSTE) case definition for salmonellosis in effect at the time of reporting, including the 2005, 2012, and 2017 definitions [18]. County-level population estimates were obtained from the U.S. Census Bureau’s American Community Survey (ACS) [19]. The University of Georgia Institutional Review Board determined that this study does not involve human subjects research. This activity was reviewed by CDC, deemed research not involving human subjects, and was conducted consistently with applicable federal law and CDC policy. See, for example, 45 C.F.R. part 46; 21 C.F.R. part 56; 42 U.S.C. §241(d), 5 U.S.C. §552a, 44 U.S.C. §3501 et seq.

### County-level covariates and urbanicity classification

County-level covariates were obtained from publicly available data sources and included demographic composition, socioeconomic factors, healthcare access, and health status. Data sources included ACS, the Small Area Income and Poverty Estimates (SAIPE) Program [20], and the University of Wisconsin Population Health Institute County Health Rankings & Roadmaps (UWPHI CHRR) [21]. All covariates and corresponding data sources are listed in Table 1. One composite measure in our analysis obtained from the UWPHI CHRR is the Food Environment Index (FEI), which is a county-level composite measure ranging from 0 (least favourable) to 10 (most favourable), based on limited access to healthy foods and food insecurity [22]. The primary outcome was the annual age-adjusted county-level incidence of *Salmonella*, expressed as cases per 100 000 population. Urban–rural classification was based on CDC’s National Center for Health Statistics (NCHS) six-level urban–rural scheme. Counties classified as large central metro, large fringe metro, medium metro, or small metro (NCHS categories 1–4) were grouped as urban, while micropolitan and noncore counties (NCHS categories 5–6) were grouped as rural. NCHS urbanicity codes are available for 2013 and 2023. The 2013 classifications were applied to NNDSS data from 2013 through 2019, and the 2023 classifications were applied to data from 2020 through 2023 [23].

**Table 1.** Data sources, county characteristics, and descriptive statistics (2011–2023) Table 1. long description.

County characteristics (2011–2023)	Data source^a^	Total (*n* = 34 797)		Urban/rural differences				
				Urban counties (*n* = 14 348)		Rural counties (*n* = 20 449)		*P*-value^c^
		Median^b^	IQR	Median	IQR	Median	IQR	
*Salmonella* incidence (per 100 000 population)	NNDSS and ACS	36.59	53.96	21.61	24.87	44.04	51.77	<0.001
Demographic composition								
Sex								
Male, %	ACS	49.60	1.60	49.40	1.30	49.80	1.80	<0.001
Female, %	ACS	50.40	1.60	50.60	1.30	50.20	1.80	<0.001
Age								
<5 years old, %	ACS	5.80	1.30	5.90	1.20	5.80	1.40	<0.001
5–15 years old, %	ACS	12.80	2.20	12.90	2.00	12.60	2.30	<0.001
15–24 years old, %	ACS	12.30	2.30	12.70	2.20	12.00	2.30	<0.001
25–44 years old, %	ACS	23.60	3.60	24.90	3.30	22.80	3.20	<0.001
45–64 years old, %	ACS	27.20	3.20	27.00	3.40	27.30	3.10	<0.001
>65 years old, %	ACS	17.30	5.40	15.50	4.90	18.50	5.10	<0.001
Race/ethnicity								
White, Non-Hispanic, %	ACS	82.80	27.70	77.70	26.10	86.40	27.30	<0.001
African American, Non-Hispanic, %	ACS	2.60	10.70	5.60	13.20	1.40	6.40	<0.001
Native American, Non-Hispanic, %	ACS	0.20	0.40	0.20	0.30	0.30	0.60	<0.001
Asian, Non-Hispanic, %	ACS	0.60	1.00	1.20	2.10	0.40	0.60	<0.001
Hispanic, all races, %	ACS	4.10	7.60	5.20	8.30	3.30	6.60	<0.001
Socioeconomic factors								
Income								
Median household income (1 000s)^d^	SAIPE	50.56	18.13	56.85	21.20	46.84	14.76	<0.001
Poverty^e^								
Poverty, %	SAIPE	14.70	7.90	13.10	7.10	15.90	8.30	<0.001
Unemployment^f^								
Unemployed, %	UWPHI CHRR	4.62	2.85	4.53	2.67	4.69	3.00	<0.001
Educational attainment								
High school, %^g^	ACS	34.80	9.90	31.40	10.40	36.60	8.50	<0.001
Bachelor’s degree or higher, %^g^	ACS	19.20	11.10	24.30	14.20	17.00	7.40	<0.001
Healthcare access								
Food environment index	UWPHI CHRR	7.60	1.40	7.80	1.30	7.40	1.40	<0.001
No. primary care physicians per 100 000 population	UWPHI CHRR	48.98	38.78	56.94	44.54	45.31	32.79	<0.001
Health status								
Poor/fair health, %	UWPHI CHRR	17.60	7.00	16.50	5.90	18.60	7.70	<0.001
Diabetic, %	UWPHI CHRR	10.90	3.40	10.50	3.00	11.30	3.50	<0.001
HIV rate per 100 000 population	UWPHI CHRR	121.90	159.74	152.70	176.20	100.10	128.60	<0.001

SD, standard deviation; IQR, interquartile range.

a Data sources: NNDSS, National Notifiable Diseases Surveillance System (https://www.cdc.gov/nndss/infectious-disease/index.html); ACS, American Community Survey (https://www.census.gov/programs-surveys/acs/data.html); SAIPE, Small Area Income and Poverty Estimates Program (https://www.census.gov/programs-surveys/saipe.html).

b Values represent the median and interquartile range (IQR) across counties. Percentages indicate the proportion of county residents with the specified characteristic.

c *P*-value calculated using Wilcoxon rank-sum test.

d Median household income is reported in thousands of U.S. dollars.

e Poverty represents the percentage of residents with family income below the federal poverty threshold defined by the U.S. Census Bureau (SAIPE).

f Unemployment is the number of people ages 16+ unemployed and looking for work (CHRR).

g Educational attainment in adults 25+.

### Statistical analysis

The annual *Salmonella* incidence per 100 000 residents was calculated for each county using intercensal population estimates from the ACS and standardized to the 2020 U.S. population. Each county contributed up to 13 annual observations (one per year from 2011 to 2023), which were treated as county-year units and pooled across years. Medians and interquartile ranges were calculated overall and separately for county–year observations classified as rural or urban. Differences in *Salmonella* incidence and county-level covariates between rural and urban counties were assessed using the Wilcoxon rank-sum test due to the non-normal nature of the distributions (Table 1). Yearly age-adjusted incidence rates with 95% confidence intervals were calculated for urban, rural, and all counties combined, and temporal patterns were visualized using LOWESS smoothing.

County-year *Salmonella* case counts were modelled using generalized linear models with a log link and the logarithm of county population as an offset. Models adjusted for the demographic, socioeconomic, healthcare-access, and health-status covariates are listed in Table 1, with standard errors clustered by state. Calendar year was included as a categorical variable in the primary national and regional models to account for year-specific variation in incidence without imposing a linear or other parametric form on the temporal pattern. Negative binomial and quasi-Poisson specifications were compared using Pearson residual diagnostics, and the model providing the better-fitting mean–variance relationship was selected [24]. Rural–urban incidence rate ratios were estimated nationally and separately by U.S. Census region, with urban counties as the reference group [6, 25]. We fitted an additional adjusted negative binomial model containing an interaction between urbanicity and categorical calendar year to evaluate whether rural–urban differences varied by year. Adjusted rural-to-urban incidence rate ratios were estimated separately for each of the 13 study years. We used a Bonferroni-adjusted threshold of 0.0038 (0.05/13) to determine statistical significance of multiple annual comparisons. A joint Wald test of the urbanicity-by-year interaction terms was used to assess whether the magnitude of the rural–urban association differed across years. Multicollinearity among covariates in the generalized linear model’s linear predictor was assessed using variance inflation factors (VIFs). Values greater than 10 were considered evidence of extreme multicollinearity and prompted evaluation of whether related variables should be removed or combined [26]. Factors contributing to rural–urban differences in age-adjusted *Salmonella* incidence were examined using Blinder–Oaxaca decomposition models [27–29] to quantify the relative contributions of demographic, socioeconomic, healthcare access, and health-related population characteristics to observed differences, both nationally and by Census region. All models were fully adjusted for covariates listed in Table 1.

Sensitivity analyses evaluated the robustness of findings by recalculating negative binomial and Blinder–Oaxaca models using an alternative urban–rural classification (NCHS categories 1–5 as urban, category 6 as rural) and by examining different time periods to assess potential impacts of NNDSS modernization in 2014 (2014–2023) and COVID-19-related reporting disruptions (2011–2019) [30–31]. All analyses were conducted in Stata BE version 17.0 (StataCorp LLC), with two-sided *P*-values <0.05 considered statistically significant. The Stata code used for all analyses is available from the corresponding author upon reasonable request.

## Results

### Differences in rural–urban characteristics and *Salmonella* incidence

**Figure 1.**
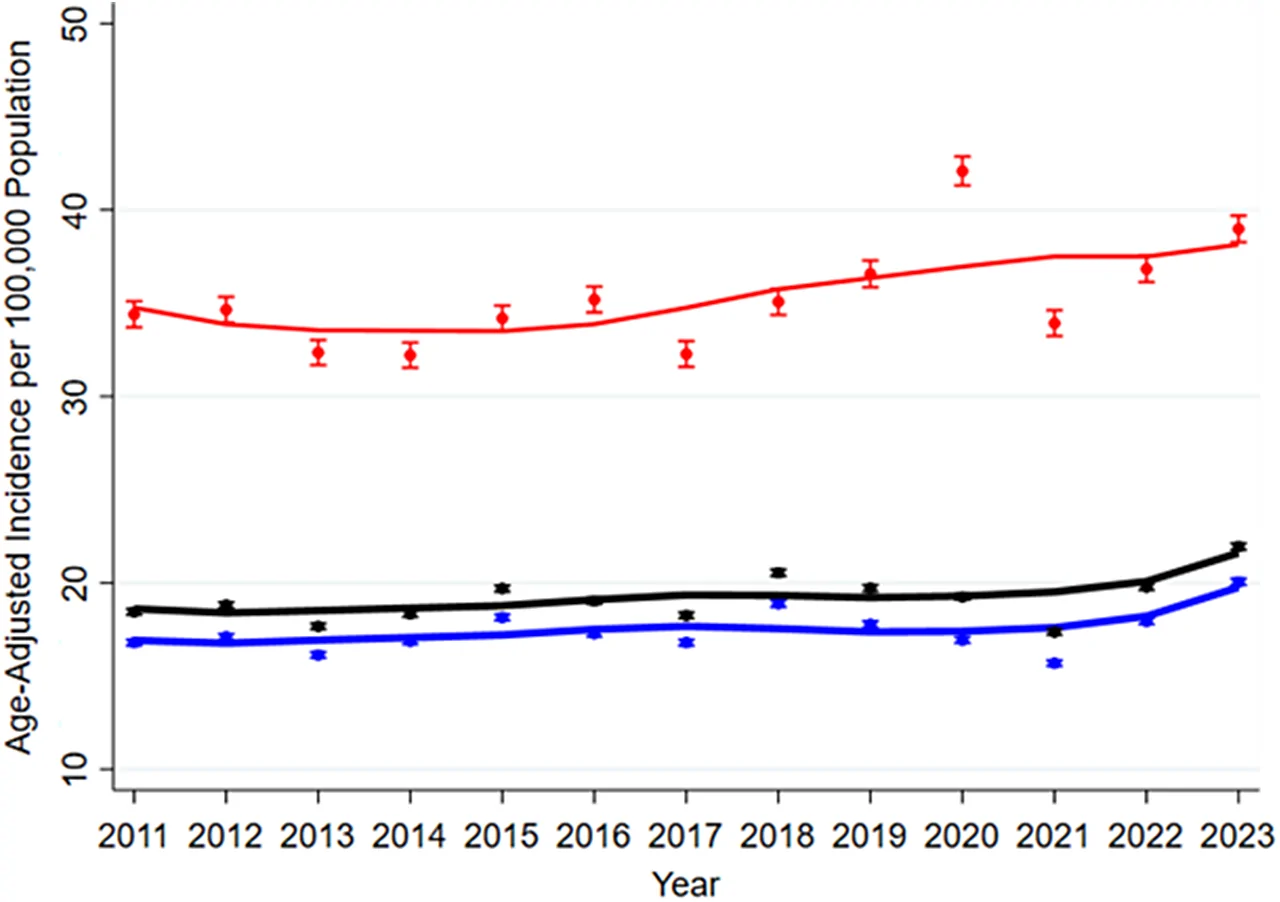
Age-adjusted *Salmonella* incidence per 100 000 population in the U.S. counties, 2011–2023, by urban, rural, and total. *Note*: Annual age-adjusted incidence rates (dots with 95% CI) of *Salmonella* from NNDSS, 2011–2023, with LOWESS smoothing curves: red = rural, blue = urban, black = all counties. Figure 1. long description.

Rural and urban counties differed descriptively across several demographic, socioeconomic, healthcare-access, and health-status characteristics included as model covariates. Compared with urban counties, rural counties had lower median household income and educational attainment, fewer primary care physicians per 100 000 population, and higher levels of poverty, poor or fair health, and diabetes. These variables were included principally as adjustment factors and to assess their contributions to the explained rural–urban difference in *Salmonella* incidence (Table 1). Age-adjusted *Salmonella* incidence, the primary outcome, was also significantly higher in rural than urban counties (*P* < 0.05). From 2011 to 2023, age-adjusted *Salmonella* incidence remained higher in rural than urban counties (Figure 1). Rural incidence generally ranged from the low 30s to nearly 40 per 100 000, while urban incidence remained between 15 and 20 per 100 000 across the study period. A notable decline occurred in 2021, after which overall incidence rose moderately, but the rural–urban gap persisted.

### Adjusted rural–urban differences in *Salmonella* incidence in the United States and by census region

Comparison of negative binomial and quasi-Poisson specifications indicated that the negative binomial model provided a better representation of the observed mean–variance relationship (Supplementary Figure 1). Pearson residuals from the negative binomial model were more tightly centred around zero and exhibited less systematic variation across fitted values than those from the quasi-Poisson model. The quasi-Poisson model also showed substantial residual overdispersion (Pearson dispersion = 9.49). We, therefore, retained the negative binomial specification for the primary analyses.

Consistent with descriptive patterns, adjusted multivariable negative binomial regression models controlling for categorical calendar year, demographic, socioeconomic, healthcare, and health-related covariates showed that rural counties across the United States had higher age-adjusted *Salmonella* incidence than urban counties. Rural counties had approximately 9% higher incidence compared with urban counties (incidence rate ratio [IRR] = 1.090, 95% CI: 1.018–1.168, *P* = 0.014). Regionally, rural counties had 14% higher incidence in the Northeast (IRR =1.137, 95% CI: 1.045–1.233, *P* < 0.001), 8% higher incidence in the Midwest (IRR =1.083, 95% CI: 1.040–1.123, *P* < 0.001), 9% higher incidence in the South (IRR =1.087, 95% CI: 1.001–1.181, *P* = 0.047), and 18% higher incidence in the West (IRR =1.176, 95% CI: 1.047–1.322, *P* = 0.006) compared to urban counties in each region (Table 2).

**Table 2.** Association between county urbanicity and *Salmonella* incidence, overall and by U.S. census region Table 2. long description.

Region	Number of jurisdictions	*Salmonella* incidence per 100 000 population (urban)	*Salmonella* incidence per 100 000 population (rural)	IRR	95% CI	*P*-value	
The United States	51	17.41	35.32	1.090	1.018–1.168	0.046	
Northeast	9	13.38	19.20	1.137	1.045–1.233	<0.001	
Midwest	12	14.41	32.56	1.083	1.040–1.123	<0.001	
South	17	23.42	44.08	1.087	1.001–1.181	0.047	
West	13	13.26	27.10	1.176	1.047–1.322	0.006	

Incidence values represent age adjusted rates per 100 000 population. Incidence rate ratios (IRRs) are adjusted estimates from negative binomial regression models controlling for categorical calendar year, sex, age, race/ethnicity, median household income, poverty, unemployment, educational attainment, food environment index, number of primary care physicians per 100 000 population, self-reported poor/fair health, diabetes prevalence, and HIV prevalence. Calendar year was modelled as a categorical variable to account for year-specific variation in incidence without imposing a linear temporal trend. Urban counties serve as the reference category.

In the additional model containing an interaction between urbanicity and categorical calendar year, adjusted rural-to-urban incidence rate ratios (IRRs) were greater than 1.0 in each study year, indicating consistently higher estimated incidence in rural counties. Year-specific adjusted rural-to-urban IRRs were not statistically significant from 2011 through 2019. Adjusted rural–urban differences were statistically significant after Bonferroni correction in 2020 (IRR = 1.199, 95% CI: 1.114–1.291, *P* < 0.001), 2021 (IRR = 1.149, 95% CI: 1.060–1.246, *P* = 0.001), 2022 (IRR = 1.137, 95% CI: 1.062–1.217, *P* < 0.001), and 2023 (IRR = 1.140, 95% CI: 1.052–1.236, *P* = 0.001) (Supplementary Table 1). The magnitude of the rural–urban association varied significantly across years (joint Wald test of the urbanicity-by-year interaction: χ^2^ = 70.14, *P* < 0.001).

### Contributions of demographic, socioeconomic, healthcare, and health-related factors to rural–urban differences

Blinder–Oaxaca decomposition suggested that observed covariates accounted for most of the national gap (66%), with the remaining 34% unexplained. Nationally, the explained portion was driven primarily by differences in the proportion of adults 65+ (≈ 60%), proportion of female residents (≈ 19%), median household income (1 000s) (≈ 14%), food environment characteristics (≈ 22%), and primary care physicians per 100 000 residents (≈ 12%). The county-level proportion of adults with a bachelor’s degree or higher contributed negatively to the explained difference (≈ −42%), indicating that its distribution was associated with a smaller rural–urban incidence gap; this ecological association should not be interpreted causally (Table 3).

**Table 3.** Blinder–Oaxaca decomposition of urban–rural differences in *Salmonella* incidence, overall and by U.S. census region Table 3. long description.

Covariate	The United States				Northeast				Midwest				South				West			
	*β*	SE	*P*-value	% Diff	*β*	SE	*P*-value	% Diff	*β*	SE	*P*-value	% Diff	*β*	SE	*P*-value	% Diff	*β*	SE	*P*-value	% Diff
Incidence (rural counties)	56.89	1.15	<0.001		22.38	0.55	<0.001		53.75	2.61	<0.001		54.48	1.21	<0.001		50.77	4.47	<0.001	
Incidence (urban counties)	28.44	0.46	<0.001		15.86	0.21	<0.001		22.61	0.35	<0.001		36.78	0.85	<0.001		17.15	0.63	<0.001	
Difference	28.45	1.24	<0.001	100	7.51	0.59	<0.001	100	31.13	2.63	<0.001	100	27.71	1.48	<0.001	100	33.63	4.52	<0.001	100
**Unexplained**	**9.76**	**1.60**	**<0.001**	34.31	**3.64**	**0.72**	**<0.001**	48.47	**1.84**	**1.99**	**0.354**	5.91	**12.54**	**2.53**	**<0.001**	45.25	**−8.72**	**13.71**	**0.525**	−25.93
**Explained**	**18.69**	**1.57**	**<0.001**	65.69	**3.87**	**0.67**	**<0.001**	51.53	**29.29**	**3.21**	**<0.001**	94.09	**15.17**	**1.60**	**<0.001**	54.75	**42.35**	**17.24**	**0.014**	125.93
**Demographic composition**																				
Female, %	5.36	0.81	<0.001	18.84	1.35	0.41	0.001	17.98	3.88	0.74	<0.001	12.46	3.69	0.87	<0.001	13.32	14.55	5.97	0.015	43.26
< 5 years old, %	−2.06	0.95	0.031	−7.24	−1.20	0.61	0.049	−15.98	−8.70	3.62	0.016	−27.95	0.72	0.60	0.235		−13.35	5.82	0.022	−39.70
5–14 years old, %	−2.14	1.03	0.038	−7.52	0.24	0.43	0.572		5.08	6.33	0.422		−2.77	0.81	0.001	−10.00	−0.91	2.83	0.749	
15–24 years old, %	−1.60	0.66	0.015	−5.62	−0.21	0.16	0.208		−2.57	1.74	0.140		−0.85	0.78	0.275		−5.87	2.86	0.040	−17.45
45–64 years old, %	1.83	0.68	0.007	6.43	0.26	0.46	0.568		4.68	2.14	0.028	15.03	0.26	0.39	0.506		12.64	6.67	0.058	
> 65 years old, %	16.97	2.13	<0.001	59.65	1.93	0.47	<0.001	25.70	35.23	3.80	<0.001	113.17	9.08	2.54	<0.001	32.77	29.19	11.40	0.010	86.80
African American, Non-Hispanic, %	−0.76	0.16	<0.001	−2.67	0.36	0.24	0.141		0.75	1.73	0.663	2.41	0.39	0.17	0.018	1.41	−0.77	1.35	0.567	
Native American, Non-Hispanic, %	−0.38	0.16	0.016	−1.34	0.17	0.12	0.158		0.16	0.43	0.713	0.51	−0.37	0.14	0.008	−1.34	−0.53	0.91	0.565	
Asian, Non-Hispanic, %	−0.47	0.40	0.235		0.58	0.25	0.020	7.72	1.78	0.95	0.061		1.51	0.67	0.025	5.45	−5.71	2.80	0.041	−16.98
Hispanic, all races, %	−0.45	0.20	0.021	−1.58	0.06	0.21	0.761		0.09	0.15	0.539		−0.24	0.16	0.153		−2.66	0.70	<0.001	−7.91
**Socioeconomic factors**																				
Median household income (1 000s)^a^	3.98	1.96	0.042	13.99	−0.20	0.42	0.641		−3.16	2.83	0.264		6.94	3.24	0.032	25.05	4.19	4.15	0.313	
Poverty, %^b^	0.29	1.13	0.795		0.24	0.27	0.028	3.20	2.42	0.93	0.009	7.77	−1.84	2.30	0.423		−6.57	2.60	0.012	−19.54
Unemployed, %^c^	−1.63	0.30	<0.001	−5.73	0.02	0.03	0.452		−1.08	0.48	0.025	−3.47	−0.48	0.40	0.221		1.07	0.52	0.039	3.18
High school, %	4.11	2.29	0.073		2.34	0.30	<0.001	31.16	−6.13	3.76	0.103		10.79	3.43	0.002	38.94	22.99	15.76	0.145	
Bachelor’s or higher, %	−12.02	3.89	0.002	−42.25	−0.17	0.73	0.814		−11.92	5.15	0.021	−38.29	−15.88	5.31	0.003	−57.31	−23.17	15.96	0.147	
**Healthcare access**																				
Food environment index	6.16	1.45	<0.001	21.65	−0.39	0.19	0.041	−5.19	5.66	2.88	0.049	18.18	5.68	1.60	<0.001	20.50	11.58	4.70	0.014	34.43
PCP rate	3.32	0.87	<0.001	11.67	−0.41	0.19	0.028	−5.46	4.05	2.30	0.078		3.57	0.71	<0.001	12.88	3.59	2.33	0.123	
**Health status**																				
Poor/fair health, %	−1.02	0.82	0.215	−3.59	−0.32	0.14	0.019	−4.26	−3.20	1.06	0.003	−10.28	−2.96	1.45	0.041	−10.68	2.04	2.14	0.342	
Diabetic, %	−1.03	0.47	0.028	−3.62	−0.16	0.08	0.049	−2.13	−0.86	0.36	0.017	−2.76	−1.72	0.91	0.059		−0.96	0.60	0.109	
HIV rate	0.23	0.50	0.642		−0.64	0.16	<0.001	−8.52	3.12	1.35	0.021	10.02	−0.35	0.42	0.404		1.00	1.00	0.316	

*β* = coefficient; SE = standard error.

Percent difference (%Diff) values are reported only for covariates with statistically significant contributions (*P* < 0.05). % Diff represents the proportion of the total urban–rural difference in age-adjusted *Salmonella* incidence explained by each covariate. Positive values indicate a contribution to higher incidence in rural counties, while negative values indicate a contribution to higher incidence in urban counties. The sum of the explained components shows the share of the overall difference attributable to observed covariates, and the unexplained component captures differences in coefficients or unmeasured factors.

a Median household income is reported in thousands of U.S. dollars.

b Poverty represents the percentage of residents with family income below the federal poverty threshold defined by the U.S. Census Bureau (SAIPE).

c Unemployment is the number of people ages 16+ unemployed and looking for work (CHRR).

The dominant contributors to rural–urban differences in *Salmonella* incidence varied by the U.S. Census region. Educational attainment was the primary driver in the Northeast and the South. The proportion of residents completing high school accounted for approximately 31% of the total rural–urban difference in the Northeast and 39% in the South; however, no formal test was conducted to determine whether these contributions differed between regions. Age accounted for a relatively large share of the estimated explained difference in the Midwest and West. In the Midwest, the contribution of adults aged ≥65 years exceeded the total explained difference (≈113%), indicating that contributions from other covariates offset part of this estimate. In the West, adults aged ≥65 years accounted for approximately 87% of the rural–urban difference. Across regions, some characteristics that were more prevalent in urban counties, including higher educational attainment and a higher proportion of young children, had negative estimated contributions, indicating that they offset part of the explained rural–urban difference. These estimates should be interpreted cautiously because regional decomposition components were not formally compared and may be sensitive to correlation among covariates. Together, these findings indicate descriptive regional heterogeneity in the estimated relative contributions of county-level characteristics, while the proportion of adults aged ≥65 years remained an important contributor nationally and across all regions. The regional decomposition estimates were derived from separate models and were not formally compared.

### Sensitivity analyses

Using alternative classifications (NCHS categories 1–5 as urban, category 6 as rural), rural counties had ≈12% higher incidence (IRR = 1.123, 95% CI: 1.050–1.214, *P* = 0.042), with ≈36% of the difference explained by covariates. By time period, incidence was 11% higher for 2011–2019 (IRR = 1.110, 95% CI: 1.018–1.209, *P* = 0.049) and 16% higher for 2014–2023 (IRR = 1.143, 95% CI: 1.067–1.221, *P* = 0.037), with ≈74% and ≈64% of the rural–urban difference explained by covariates, respectively (Supplementary Table 2). Overall, these analyses confirm that the rural–urban differences in *Salmonella* incidence are robust across definitions and time periods.

## Discussion

In this study, age-adjusted *Salmonella* incidence, after adjusting for other factors, was 8% higher in the Midwest, 9% higher in the South, 14% higher in the Northeast, and 18% higher in the West. These findings suggest a persistent rural–urban disparity that varies by region. Prior research provides mixed evidence. A recent study found that higher population density increases the likelihood of reporting ≥1 *Salmonella* case, possibly reflecting differences in detection and reporting rather than population-adjusted incidence rates; however, rural counties that do report cases are more likely to experience higher *Salmonella* incidence [8]. Descriptive analyses of 1993–2002 surveillance data showed higher *Salmonella* incidence in urban counties, although multivariate adjustment suggested lower incidence in urban areas [5]. These differences likely reflected surveillance practices prior to the widespread adoption of culture-independent diagnostic testing and temporal shifts in the geographic distribution of disease burden [14, 32–33]. Previous studies in other states have yielded mixed results, including Michigan, where no differences were observed between urban and rural populations, and Florida, where more densely populated areas had higher reported salmonellosis incidence. This demonstrates that the relationship between urbanization and salmonellosis may vary depending on the location [11–14]. These findings highlight the need for additional research to clarify why reported *Salmonella* incidence differs between rural and urban counties. Potential explanations include geographic variation in surveillance and diagnostic practices, food consumption and availability, food purchasing environments, and consumers’ food-safety knowledge, attitudes, and practices.

We found that county-level factors explained most of the rural–urban gap. Nationally, the largest contribution to the rural–urban gap resulted from differences in age distribution across these groups, reflecting high representation of adults aged 65 years and older in rural counties. This finding is consistent with previous evidence indicating that older adults are more likely than others to develop severe illness and present laboratory-confirmed *Salmonella* infection [3–4]. Sex distribution accounted for a secondary share of the rural–urban differences, consistent with ecological studies reporting modest or inconsistent associations between county-level sex composition and *Salmonella* incidence [5, 10, 11, 13, 34]. In addition, socioeconomic and structural factors played a part, with education, food environment, health status, and primary care access contributing to a significant degree to the rural–urban gap. County-level educational attainment was associated with the explained rural–urban difference, but the ecological design does not establish that increasing educational attainment in rural counties would reduce *Salmonella* incidence. Lower educational attainment may also partly reflect longstanding selective outmigration of college-educated young adults from rural communities, a pattern that can contribute simultaneously to lower county-level educational attainment and older population age structures [35]. Educational attainment should not be interpreted as a direct measure of food safety knowledge or food handling behaviours, which may vary independently of formal education. Although prior studies have identified several of these factors as correlates of *Salmonella* incidence, no studies have examined or quantified their contribution to rural–urban differences. These findings identify potential areas for intervention research, including prevention strategies for older adults, improved access to primary care, programmes addressing barriers to healthy food access, and investments in rural surveillance and public health infrastructure. Pilot studies and implementation evaluations are needed to determine whether these approaches are feasible and effective in reducing reported *Salmonella* incidence in rural communities.

Rural–urban differences in *Salmonella* incidence varied across Census regions, reflecting regional differences in population demographics, healthcare access, food environment characteristics, and other structural characteristics. Reported *Salmonella* incidence was higher in rural counties than in urban counties in all four regions of the United States. Although the direction of the rural–urban association was consistent across all four regions, the magnitude of the difference and the estimated contributions of county-level characteristics varied regionally. This heterogeneity suggests that the factors underlying higher reported incidence in rural counties may differ across geographic settings. The descriptive regional variation in estimated contributions may help guide future research priorities. Studies in the Northeast and South could examine how educational attainment, food access, food-handling practices, and related community conditions are associated with reported incidence. Studies in the Midwest and West could investigate whether age-related clinical, behavioural, residential, or surveillance factors contribute to the observed patterns. The ecological findings do not establish that interventions targeting these characteristics would reduce *Salmonella* incidence or narrow the rural–urban difference. In the Northeast, Midwest, and South, the large unexplained components suggest that unmeasured factors such as agricultural exposures and food production or handling practices may be influencing *Salmonella* incidence [36–39]. Non-foodborne pathways may also contribute to the unexplained rural–urban difference. Beshearse et al. estimated that 66% of domestically acquired non-typhoidal *Salmonella* illness was foodborne, with the remainder attributed to waterborne, person-to-person, animal-contact, and environmental transmission [40]. The relative importance of these pathways may differ between rural and urban counties, including through differences in private-water-system use, livestock and other animal contact, and environmental exposures. Additional research incorporating surveillance practices, diagnostic access, agricultural exposures, environmental conditions, water sources, food-handling behaviours, and other individual- and community-level factors is needed to explain the remaining rural–urban differences. Such research could inform the development and evaluation of appropriately tailored population- or community-level prevention strategies.

## Limitations

This study has several limitations. First, the analysis used county-level data from NNDSS, a passive surveillance system that may underreport cases or vary in reporting across counties, particularly in rural areas with limited laboratory capacity. NNDSS data used in this analysis did not include *Salmonella* serotype information for serotype-specific analyses. Given that epidemiological patterns differ between serotypes, it is possible that the combination of all serotypes may have concealed the disparities among them with regard to geographic location, reservoir, and mode of transmission [5]. Second, county-level relationships cannot be assumed to reflect individual-level relationships, and the decomposition findings do not prove that altering a contributing factor at the county level will alter Salmonella prevalence. Individual-level factors such as food intake and preparation behaviours, exposure to animals, drinking water, access to health care, diagnostics, and underlying clinical vulnerability may be involved. Third, our measure of urban–rural status was based on NCHS’s 2013 and 2023 definitions, which may not fully reflect changes in population density between 2011 and 2023. Fourth, although the Blinder–Oaxaca decomposition estimates how much of the rural–urban difference is explained by measured factors, it cannot capture interactions between variables or unmeasured factors, leaving some differences unexplained. The analysis did not include potentially relevant agricultural and water-source characteristics, such as livestock and poultry production or reliance on private wells. Consistent annual county-level measures were unavailable across the full study period: nationally comprehensive agricultural data are collected every 5 years, and national private-well estimates are periodic rather than annual. Using intermittent estimates across multiple years could have introduced exposure misclassification and may have contributed to the unexplained rural–urban difference. Finally, temporal patterns may be affected by changes in surveillance practices, case definitions, and reporting completeness over time, including potential disruptions during the COVID-19 pandemic.

## Advantages

NNDSS provided the combination of nationwide geographic coverage, county-level case information, and annual observations needed to link reported *Salmonella* incidence with county-level demographic, socioeconomic, healthcare, and health-status covariates. This combination made NNDSS particularly well suited to a longitudinal national analysis of rural–urban differences. This study employs several publicly available county-level data sources to explore how demographic, socioeconomic, and health-related factors contribute to *Salmonella* incidence. The decomposition analysis identified explained and unexplained parts of the rural–urban difference, while regional breakdowns showed variation in both the size of the difference and its causes, supporting region-specific public health planning. Taken together, these strengths improve the study’s capacity to guide targeted surveillance, prevention efforts, and resource allocation to address rural–urban differences in salmonellosis.

## Conclusions

This national-level analysis reveals the persistence of the rural–urban difference in *Salmonella* incidence in the United States, with higher rates in rural counties driven primarily by demographic, socioeconomic, and healthcare-related factors. Although many of the rural–urban differences were explained by county characteristics, the extent of these differences varied across regions, highlighting substantial regional heterogeneity. These findings identify priorities for further research into the individual- and community-level mechanisms underlying rural–urban differences, including educational and food-environment characteristics, age-related factors, surveillance and diagnostic practices, and environmental and agricultural exposures. Evidence from such studies will be needed to develop and evaluate appropriately tailored prevention strategies.

## Supporting information

10.1017/S0950268826102192.sm001 Joseph et al. supplementary material 1 Joseph et al. supplementary material

10.1017/S0950268826102192.sm002 Joseph et al. supplementary material 2 Joseph et al. supplementary material

## Data Availability

The data supporting the findings of this study were obtained from the National Notifiable Diseases Surveillance System (NNDSS), the U.S. Census Bureau American Community Survey (ACS) the Small Area Income and Poverty Estimates (SAIPE) Program, and the University of Wisconsin Population Health Institute County Health Rankings & Roadmaps (CHRR). Publicly available NNDSS data can be accessed through the CDC, and the ACS, SAIPE, and CHRR data are available from their respective public data sources. The NNDSS case-level data used for this analysis were accessed through CDC’s System for Enteric Disease Response, Investigation, and Coordination (SEDRIC) and are not redistributed by the authors. Stata code used for the analyses is available from the corresponding author upon reasonable request.

## Supplementary material

The supplementary material for this article can be found at http://doi.org/10.1017/S0950268826102192.

## Disclaimer

This link is on a development site and not yet published publicly. The publication criteria document summarizes which case classification statuses meet the print criteria individually for 2016–2023 – Notice to Data Users and Publication Criteria | National Notifiable Diseases Surveillance System (NNDSS) | CDC. The findings and conclusions in this report are those of the authors and do not necessarily represent the official position of the Centers for Disease Control and Prevention. Use of trade names and commercial sources is for identification only and does not imply endorsement by the Centers for Disease Control and Prevention, the Public Health Service, or the U.S. Department of Health and Human Services.

## Long descriptions

### Table 1. Long description

Table 1 summarizes data sources and descriptive statistics for county characteristics from 2011–2023, overall and by urban and rural status. Characteristics include Salmonella incidence, demographics, socioeconomic factors, educational attainment, healthcare access, and health status. Values are reported as medians and interquartile ranges. All urban-rural comparisons, assessed using Wilcoxon rank-sum tests, were statistically significant at P less than 0.001.

Navigate back to Table 1..

### Table 2. Long description

Table 2 compares age-adjusted Salmonella incidence in urban and rural counties for the United States overall and across the Northeast, Midwest, South, and West. Rural counties had higher Salmonella incidence than urban counties in every region. Adjusted incidence rate ratios were greater than 1 for all regions, with statistically significant associations between county urbanicity and Salmonella incidence.

Navigate back to Table 2..

### Table 3. Long description

Table 3 decomposes urban-rural differences in age-adjusted Salmonella incidence for the United States overall and for the Northeast, Midwest, South, and West. It separates the total difference into explained and unexplained components and shows the contribution of demographic, socioeconomic, healthcare access, and health-status covariates. Observed covariates explained 65.69% of the national urban–rural difference, with regional variation in the proportion explained.

Navigate back to Table 3..

### Figure 1. Long description

The x-axis is labeled Year and ranges from 2011 to 2023 in annual increments. The y-axis is labeled Age-Adjusted Incidence per 100,000 Population and ranges from 10 to 50. Three distinct data series are plotted with dots representing annual rates, vertical error bars for 95 percent C I, and L O W E S S smoothing curves.

* The top red curve represents rural counties. It starts at approximately 35 in 2011, dips slightly until 2014, and then shows a steady upward trend, reaching nearly 40 by 2023. A significant outlier peak appears in 2020 at approximately 42.

* The middle black curve represents all counties. It remains relatively stable between 18 and 20 from 2011 to 2021, before showing a slight increase toward 22 in 2023.

* The bottom blue curve represents urban counties. It follows a similar trajectory to the black curve but at a lower incidence level, starting near 17 and ending near 20 in 2023.

Across the entire period, rural incidence rates are consistently and significantly higher than urban and total population rates.

Navigate back to Figure 1..
