# Supplementary material for: County-level differences in Salmonella incidence across urban and rural United States, 2011–2023
Source: Epidemiol Infect. 2026 Oct 14;154:e134. doi: 10.1017/S0950268826102192 (PMC13749191; doi:10.1017/S0950268826102192)
Supplement: Supplementary file 1 [file S0950268826102192sup001.docx]

**Supplemental Figure 1**

**A**

**B**
